# Clonal diversity and detection of carbapenem resistance encoding genes among multidrug-resistant *Acinetobacter baumannii* isolates recovered from patients and environment in two intensive care units in a Moroccan hospital

**DOI:** 10.1186/s13756-017-0262-4

**Published:** 2017-09-26

**Authors:** Jean Uwingabiye, Abdelhay Lemnouer, Ignasi Roca, Tarek Alouane, Mohammed Frikh, Bouchra Belefquih, Fatna Bssaibis, Adil Maleb, Yassine Benlahlou, Jalal Kassouati, Nawfal Doghmi, Abdelouahed Bait, Charki Haimeur, Lhoussain Louzi, Azeddine Ibrahimi, Jordi Vila, Mostafa Elouennass

**Affiliations:** 1 Department of Clinical Bacteriology, Mohammed V Military Teaching Hospital, Research Team of Epidemiology and Bacterial Resistance, Faculty of Medicine and Pharmacy, Mohammed V University, Rabat, Morocco; 2 Department of Clinical Microbiology and ISGlobal- Barcelona Ctr. Int. Health Res. CRESIB, Hospital Clínic - Universitat de Barcelona, Barcelona, Spain; 3 Medical Biotechnology Laboratory (Medbiotech), Faculty of Medicine and Pharmacy, Mohammed V University, Rabat, Morocco; 4 Department of Intensive Care Units , Mohammed V Military Teaching Hospital, Faculty of Medicine and Pharmacy, Mohammed V University, Rabat, Morocco

**Keywords:** *Acinetobacter Baumannii*, Multidrug-resistant, Pulsed-field gel electrophoresis, OXA genes, bla_NDM-1_ gene, Intensive care unit

## Abstract

**Background:**

Carbapenem-resistant *Acinetobacter baumannii* has recently been defined by the World Health Organization as a critical pathogen. The aim of this study was to compare clonal diversity and carbapenemase-encoding genes of *A. baumannii* isolates collected from colonized or infected patients and hospital environment in two intensive care units (ICUs) in Morocco.

**Methods:**

The patient and environmental sampling was carried out in the medical and surgical ICUs of Mohammed V Military teaching hospital from March to August 2015. All *A. baumannii* isolates recovered from clinical and environmental samples, were identified using routine microbiological techniques and Matrix-Assisted Laser Desorption/Ionization Time-of-Flight Mass Spectrometry. Antimicrobial susceptibility testing was performed using disc diffusion method. The carbapenemase-encoding genes were screened for by PCR. Clonal relatedness was analyzed by digestion of the DNA with low frequency restriction enzymes and pulsed field gel electrophoresis (PFGE) and the multi locus sequence typing (MLST) was performed on two selected isolates from two major pulsotypes.

**Results:**

A total of 83 multidrug-resistant *A. baumannii* isolates were collected: 47 clinical isolates and 36 environmental isolates. All isolates were positive for the *bla*_*OXA51-like*_ and *bla*_*OXA23-like*_ genes. The coexistence of *bla*_*NDM-1*_ */bla*_*OXA-23-like*_ and *bla*_*OXA 24-like*_ */bla*_*OXA-23-like*_ were detected in 27 (32.5%) and 2 (2.4%) of *A. baumannii* isolates, respectively. The environmental samples and the fecally-colonized patients were significantly identified (*p* < 0.05) as the most common sites of isolation of NDM-1-harboring isolates. PFGE grouped all isolates into 9 distinct clusters with two major groups (0007 and 0008) containing up to 59% of the isolates. The pulsotype 0008 corresponds to sequence type (ST) 195 while pulsotype 0007 corresponds to ST 1089.The genetic similarity between the clinical and environmental isolates was observed in 80/83 = 96.4% of all isolates, belonging to 7 pulsotypes.

**Conclusion:**

This study shows that the clonal spread of environmental *A. baumannii* isolates is related to that of clinical isolates recovered from colonized or infected patients, being both associated with a high prevalence of the *bla*_*OXA23-like*_ and *bla*_*NDM-1*_genes. These findings emphasize the need for prioritizing the bio-cleaning of the hospital environment to control and prevent the dissemination of *A. baumannii* clonal lineages.

**Electronic supplementary material:**

The online version of this article (10.1186/s13756-017-0262-4) contains supplementary material, which is available to authorized users.

## Background

Multidrug-resistant (MDR) *Acinetobacter baumannii* is recognized to be responsible for nosocomial outbreaks in severely ill patients and it is predominantly isolated in intensive care units (ICUs) around the world [1]. This microorganism colonizes certain areas of the body such as the skin, the oropharynx and the gastrointestinal tract [1]. The prevalence of digestive tract colonization varies from 8.3 to 41% in ICU patients [2, 3] but this pathogen is also the causative agent of serious infections including pneumonia, septicemia, urinary tract infection, wound infection and meningitis with mortality rates varying from 7.8 to 75% [1]. Risk factors for *Acinetobacter* colonization and infection are linked to the presence of underlying disease, long-term hospitalization, ICU stay, administration of broad spectrum antibiotics and invasive procedures such as mechanical ventilation or catheters [1, 4]. This bacterium displays an outstanding ability to survive in the environment, with some studies reporting up to 48% of environmental samples being contaminated with *Acinetobacter* [5, 6]*.* Environmental sites most likely to be contaminated include bed sheets, bed railings, touch pads of ventilator equipment, trolleys, surfaces of respiratory monitors as well as the hands and uniforms of healthcare workers [5–7].

*A. baumannii* has also the capacity to develop resistance to multiple antibiotics, which limits the therapeutic options to treat these infections [1, 4]. A recent Moroccan study showed that the resistance rate of *Acinetobacter* isolates to ciprofloxacin, imipenem, amikacin netilmicin, and colistin was 87%, 86%, 52%, 33% and 1.7%,respectively [8]. Resistance to carbapenems among *A. baumannii* isolates all over the world is mostly linked to the carriage of the *bla*_OXA-23-like,_ *bla*_OXA-24-like_, and *bla*_OXA-58-like_ genes, encoding carbapenem hydrolyzing class D β-lactamases (OXA-type), but also to the recent dissemination of the *bla*_NDM_ gene, encoding a class B metallo-β-lactamase [9–14]. Since 2010, NDM-producing *A. baumannii* isolates have been found in different countries including Kenya, Ethiopia, Algeria, Egypt, Germany, France, Spain, Turkey, India,Vietnam, China and Nepal [14, 15].

Overall, the clonal dissemination of carbapenem-resistant *A. baumannii* isolates has been documented in different countries [6, 7, 9–11] but only a few studies have focused on the clonal relationships between clinical and environmental isolates [5, 16, 17].

To our knowledge, there are no previous studies regarding the prevalence of carbapenemase encoding genes or the clonal diversity of *A. baumannii* isolates in Morocco.

The objective of this study was to characterize the carbapenemase-encoding genes and molecular diversity of clinical and environmental carbapenem-resistant *A. baumannii* isolates recovered from two ICUs of a Moroccan hospital.

## Methods

This study was carried out in the clinical bacteriology laboratory of Mohammed V Military teaching hospital in collaboration with Barcelona Institute for Global Health (IS global)-Hospital Clínic, Universitat de Barcelona.

### Sampling strategies

The patient and environmental sampling was carried out from March to August 2015 in the medical and surgical ICUs of Mohammed V Military teaching hospital, a teaching hospital with 700-beds, located in Rabat in the Kingdom of Morocco, and which contains 2 ICUs (medical and surgical) with 10 beds each, a center for burns, surgical and medical units, and laboratory and imagery departments.

The clinical isolates were recovered from the mouth, the anal margin and the groin for colonized patients and from the respiratory tract and blood cultures for infected patients. The criteria of colonization or infection were assessed according to the Centers for Disease Control and Prevention guidelines [18]. Screening samples were collected at the time of ICU admission and weekly during hospitalization. Collected clinical data included demographic characteristics, hospital wards, underlying diseases, invasive procedures, specimen types, antibiotic use, ICU length of stay and clinical outcome.

Environmental samples were collected from the patients’ rooms. At each site, an area of 10 cm^2^ was sampled using a sterile swab moistened with physiological saline [19, 20]. The sampled sites were: floors, bed sheets, medical ventilators, pillows, monitors, patient trolleys and intravenous solution stand.

All swabs were then immersed in brain heart infusion broth, incubated overnight at 37 °C and further subcultured on bromocresol purple lactose agar for the isolation of *Acinetobacter*.

All *Acinetobacter spp.* isolates were identified using routine microbiological techniques (direct examination, biochemical test of orientation, API20NE) and species identification was confirmed by matrix-assisted laser desorption/ionization time-of-flight mass spectrometry (MALDI-TOF-MS) [21].

### Antibiotic susceptibility testing

Antimicrobial susceptibility testing was performed by the disc diffusion method on Mueller-Hinton agar plates in accordance with the French Society of Microbiology in their 2015 recommendations guidelines. MDR *A. baumannii* isolates were defined as resistant to three or more classes of antibiotics represented by piperacillin/tazobactam,ceftazidime, imipenem, ciprofloxacin, aminoglycosides and colistin [22].

### PCR assays for detection of carbapenemase-encoding genes

DNA extractions from overnight cultures were performed using PureLink® Genomic DNA Kit (Invitrogen, Carlsbad, USA) and DNA IQ™ System (Promega Corporation, Madison, WI, USA) according to the manufacturer’s instructions. PCR analysis was carried out as described previously [23, 24] using the thermocycler (Biometra, Göttingen, Germany) with the primers listed in Table 1.

**Table 1:** Primers used for amplification of carbapenemase genes [23, 24]

Primer	Sequence (5′→3′)	Amplicon size (bp)
OXA-51	F: TAATGCTTTGATCGGCCTTG R: TGGATTGCACTTCATCTTGG	353
OXA-23	F: GATCGGATTGGAGAACCAGA R: ATTTCTGACCGCATTTCCA	501
OXA-24	F: GGTTAGTTGGCCCCCTTAAA R: AGTTGAGCGAAAAGGGGATT	246
OXA-58	F: AAGTATTGGGGCTTGTGCTG R: CCCCTCTGCGCTCTACATAC	599
NDM-1	F:CATTTGCGGGGTTTTTAATG R:CTGGGTCGAGGTCAGGATAG	998

Multiplex PCR assays were used to detect four carbapenemase-encoding genes (*bla*_OXA-51-like_, bla_OXA-23-like_, bla_OXA-24-like_ and bla_OXA-58-like_). PCR amplifications for bla_OXA_ genes were performed in a final volume of 50 μl, reaction mixtures contained 5 μl of 10× PCR buffer, 25 mmol/μL of MgCI2, 2.5 Mm of deoxynucleoside triphosphates (dNTPs), 0.5 μl of each primer, 1 U Taq DNA polymerase (New England BioLabs Inc., Beverly, MA, USA) and 3 μl of DNA template. The amplification conditions were initial denaturation at 94 °C for 5 min, followed by 30 cycles of 94 °C for 25 s, 52 °C for 40s and 72 °C for 50s, with final extension for 6 min at 72 °C.

Uniplex PCR was used for detection of NDM-1 gene. PCR reaction for *bla*_NDM-1_ gene was carried out by adding 0.5 μl of each primer, 5 μl of 10Xbuffer, 3 μl of MgCl_2_, 1.25 μl of dNTP’s, and 0.25 μl of Taq polymerase in a final volume of 50 μl. PCR conditions were as follows: initial denaturation at 94 °C for 10 min, followed by 32 cycles consisting of denaturation at 94 °C for 30 s, 40 s annealing at 57 °C, 50 s extension at 72 °C, followed by a final extension step at 72 °C for 5 min.

Hyperladder 100 bp (Bioline, London, UK) was used as a molecular weight marker. PCR amplification products were analyzed by gel electrophoresis in a 1.5% *w*/*v* Agarose gel stained with SYBR safe.

### Molecular typing using pulsed-field gel electrophoresis

The clonal relationship of all isolates was analyzed by PFGE as previously described with minor modifications [25]. An overnight culture on blood agar was suspended in 120 μl of cell suspension buffer (100 mM Tris-HCl, 100 mM EDTA, pH 8.0) and then, the bacterial suspension was mixed with an equal volume of 2% InCert™ Agarose (Lonza,Rockland,ME,USA) and dispensed in a plug mould. Genomic DNA in agarose plugs was lysed in the cell lysis solution (50 Mm Tris-HCl, 1% sarcosil, 100 μg/ml proteinase K), washed and digested with ApaI (New England BioLabs Inc., Beverly, MA, USA). Electrophoresis was performed in 1% InCert™ Agarose (Lonza, Rockland,ME,USA) and 0.5X TBE Buffer (PH 8.0) containing 0.02 g of thiourea using either a CHEF-DR III system (Bio-Rad Laboratories) or a CHEF-Mapper TM apparatus (Bio-Rad Laboratories) at 6 V/cm2 with switch times ranging from 5 s to 35 s at an angle of 120°, at temperature of 14 °C, for 20 h.

A standard molecular weight Lambda DNA ladder (Bio-Rad Laboratories) was included at least twice per gel to allow normalization of all fingerprints. The InfoQuest™FP v.4.5 software (Bio-Rad Laboratories) was used for dendrogram construction by the UPGMA (Unweighted Pair Group Method with Arithmetic Mean) method, based on Dice’s similarity coefficient. Isolates were considered to belong to the same PFGE cluster (pulsotype) if their Dice similarity index was ≥ 85% [26].

On the basis of the number of isolates, PFGE pulsotypes were divided into major pulsotypes (more than ten isolates/PFGE types), intermediate pulsotypes (five to nine isolates/PFGE types) and minor pulsotypes (less than five isolates/PFGE types) (Table 2).

**Table 2:** Distribution of PFGE pulsotypes according to the source of samples and status of pulsotypes

PFGE pulsotype	Clinical isolates (*N* = 47)	Environmental isolates (*N* = 36)	Total (*N* = 83)	Status of pulsotypes
	N (%)	N (%)	N (%)	
0001	7(14.9)	1(2.8)	8(9.6)	Intermediate pulsotype
0002	1(2.1)	2(5.6)	3(3.6)	Minor pulsotype
0003	1(2.1)	4(11.1)	5(6)	Intermediate pulsotype
0004	2(2.4)	0	2(4.3)	Minor pulsotype
0005	3(6.4)	4(11.1)	7(8.4)	Intermediate pulsotype
0006	1(2.1)	0	1(1.2)	Minor pulsotype (Singleton)
0007	16(34)	3(8.3)	19(22.9)	Major pulsotype
0008	14(29.8)	16(44.4)	30(36.1)	Major pulsotype
0009	2(4.3)	6(16.7)	8(9.6)	Intermediate pulsotype

### Multi locus sequence typing (MLST)

The whole-genome sequencing was performed on the extracted genomic DNA of the two selected strains of major pulsotypes by using the Nextera XT DNA library preparation kit (Illumina), with dual indexing adapters, and sequenced using an Illumina MiSeq sequencer with a 2 × 251-bp paired-end configuration. The Next Generation Sequencing Data (FASTA format) were then used for further MLST analysis, carried out using MLST Oxford scheme (https://pubmlst.org/abaumannii/).

### Statistical analysis

Statistical analysis was performed using SPSS Statistics for Windows, version 10.0. The results were expressed as effective and percentages for qualitative variables and as mean (standard deviation) or median (interquartile range: IQR) for quantitative variables. The chi-square and Fisher exact tests were used to compare the qualitative variables. A comparison between the two quantitative variables was performed using the Mann–Whitney U test for non-normal distributed variables, whereas Student’s *t*-test was used for normally distributed variables. *P* values less than 0.05 were considered significant.

## Results

### Bacterial isolates and epidemiological data

A total of 83 non-duplicate *A. baumannii* isolates were collected: 47 clinical isolates from 40 colonized or infected patients and 36 isolates from 72 environmental specimens.

Among 40 patients, 32 (80%) were colonized and 8 (20%) were infected. The epidemiological and clinical characteristics of patients are shown in Table 3. The average age was 54.38 ± 15.22 years and 30 (75%) were males representing a sex ratio M/F of 3:1. The crude mortality rate was 54.2%. The crude mortality rate was 43.8% in colonized patients and 87.5% in infected ones (*p* = 0.031). Clinical isolates were recovered from 39 screening samples including anal margin (22/47 = 46.8%), mouth (10/47 = 21.3%), groin (7/47 = 14.9%) and from 8 diagnosis samples composed of blood culture (3/47 = 6.4%), protected distal sampling (3/47 = 6.4%) and bronchial aspiration (2/47 = 4.2%).

**Table 3:** Epidemiological and clinical features of colonized or infected patients

Variable	Total *N* = 40
Number of Male patients(%)	30(75)
Mean Age (years) (Mean ± Standard deviation)	52.45 ± 16.5
Median length of ICU stay (days) [IQR]	10 [5–16]
Patients with lenght of ICU stay ≥7 days (%)	26(65)
Median duration of ICU stay prior to Colonization/infection(days) [IQR]	6[1.5–16.5]
Respiratory distress	7(17.5)
postoperative care	10(25)
Cerebrovascular accidents	13(32.5)
Severe polytrauma	6(15)
Underlyning disease	N (%)
Diabetes	7(17.5)
Chronic renal failure	5(12.5)
Arterial hypertension	6(15)
Chronic heart failure	7(17.5)
Chronic obstructive pulmonary disease	6(15)
Chronic smoking	6(15)
Solid tumor	7(17.5)
Invasive procedure	N (%)
Venous catheter	11(27.5)
Arterial catheter	4(10)
Urinary catheter	26(65)
Mechanical ventilation	24(60)
Nasogastric tube	3(7.5)
Abdominal drain	4(10)
Recent surgery	4(10)
Parenteral nutrition	30(77.5)
Dialysis	3(7.5)
Septic shock N (%)	14(76)
Previous antibiotic treatment N (%)	35(87.5)
Amoxicillin/clavulanic acid N (%)	10(25)
Ceftriaxone N (%)	7(17.5)
Imipenem N (%)	23(57.5)
Aminoglycosides N (%)	19(47.5)
Colistin	9(22.5)
Ciprofloxacin	3(7.5)
Corticosteroid therapy N (%)	19(47.5)
Death rate N (%)	21(52.5)

*ICU* Intensive care unit, *IQR* Interquartile rang

Of the 72 environmental samples, 36 (50%) yielded *A. baumannii* isolates. Surgical ICU samples were more contaminated (22/31 = 71%) than those from the medical ICU (14/41 = 34.1%) (*p* = 0.004). The environmental *A. baumannii* isolates were obtained from bed sheets (14/36 = 38.9%), floors (13/36 = 36.1%), medical ventilators (4/36 = 11.1%), patient trolleys (2/36 = 5.5%), pillows (1/36 = 2.8%), monitors (1/36 = 2.8%), and intravenous solution stands (1/36 = 2.8%).

### Antimicrobial susceptibility profile

All isolates were MDR. The difference in resistance rates between the clinical isolates and the environmental ones was not statistically significant except for gentamicin (85.1% vs 100% respectively, *p* = 0.015).

### Distribution of carbapenemase genes

The intrinsic chromosomally encoded *bla*_OXA-51 like_ gene characteristic of *A. baumannii* was detected in all isolates. All isolates were positive for *bla*_OXA-23-like_.The coexistence of *bla*_NDM-1_ with *bla*_OXA-23-like_ and *bla*_OXA 24-like_ with *bla*_OXA-23_ was detected in 27 (32.5%) and 2 (2.4%) of *A. baumannii* isolates, respectively. The *bla*_OXA-58-like_ gene was not detected. The percentage of NDM-1 carriage was significantly higher in environmental isolates than in clinical ones (18/36 = 50% vs 9/47 = 19.1%, *p* = 0.004). NDM-1 producing clinical isolates were recovered from: anal margin (88.9%) and mouth (11.1%). The NDM-1 harboring isolates were significantly (*p* = 0.006) more frequently isolated from anal margin samples than from other clinical specimens. The distribution of NDM-1 positive isolates among hospital environment samples is as follows: bed sheets (38.9%), floor (16.7%), medical ventilators (16.7%), intravenous solution stands (5.5%), monitors (5.5%), patient trolleys (5.5%) and pillows (5.5%). No significant differences (*p* = 0.432) in the number of NDM-1 positive isolates were found between different environmental sampling sites.

### PFGE and MLST analyses

**Fig. 1:**
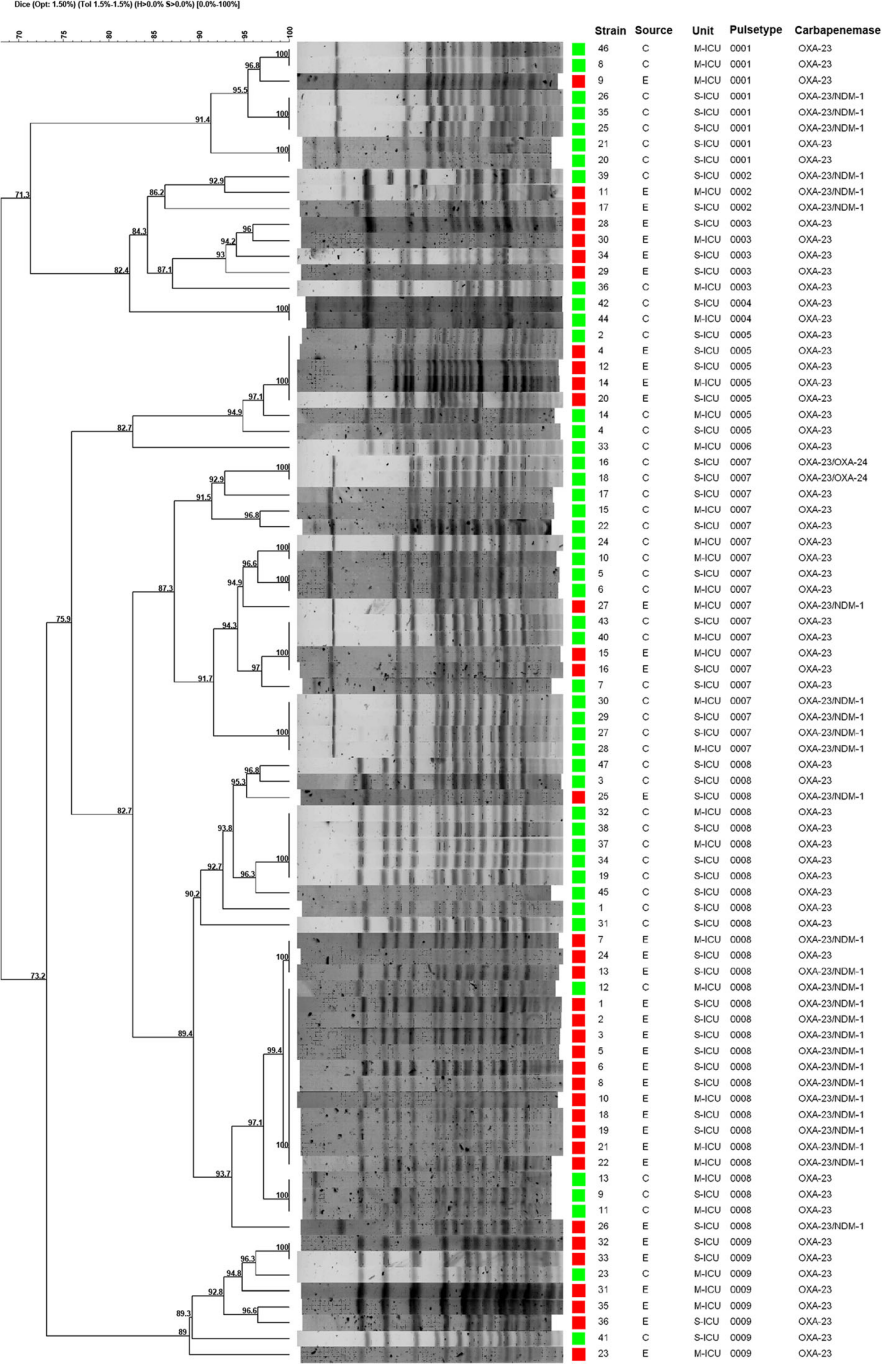
PFGE dendrogram of Clinical and environmental *A.baumannii* isolates (M-ICU: Medical intensive care unit, S-ICU: Surgical intensive care unit)

The isolates were classified into 9 PFGE pulsotypes (0001–0009) with two major pulsotypes (0008, 0007), containing up to 59% of all isolates. The strains of pulsotype 0008 belonged to sequence type (ST) 195 while those of pulsotype 0007 belonged to ST 1089. Clinical isolates were found in all 9 different pulsotypes while environmental isolates were only present in 7 pulsotypes, as they were missing from pulsotypes 0004 and 0006 which included just 2 and 1 isolates, respectively (Table 2) (Fig. 1) (Additional file 1) .

## Discussion

In the present study we have analyzed the clonal relatedness and resistance characteristics of *A. baumannii* isolates recovered. The clinical isolates were commonly isolated from the anal margin (46.8%) followed by the mouth (24%). The digestive tract of patients hospitalized in the ICU, has been identified as an important site for Acinetobacter colonization which can lead to severe infections, with a prevalence of 8.3% in Saudi Arabia [2] and 41% in Spain [3]. A study conducted in Spain by Corbella et al. showed that MDR *A. baumannii* infections occurred more frequently in patients with fecal colonization than in those without fecal colonization [3]. The crude mortality found in this study (50%) was comparable to that reported in colonized or infected patients in Italy (58%) [27] but significantly higher than that of Spanish hospitals (18.9%) [4].The mortality rate of patients depends on clinical significance of *A. baumannii.* In the current study, the mortality rate was significantly higher in infected patients than in colonized ones (87.5% vs 43.8%, *p* = 0. 031). This result is similar to that of Rodríguez-Baño J et al. in Spain who showed that crude mortality was higher in infected (27%) than in colonized patients (10%) [4].

Our findings show a higher environmental contamination around infected or colonized patients in our ICU. The presence of *A. baumannii* among environmental specimens (50%) was higher than that observed in similar studies: 7.7% in Algeria [7], 9.9% in the United States of America [19], 13.1% in China [16] and between 2% and 18% in two different studies in Turkey [5, 6]. This can be attributed to the lack of hospital decontamination procedures and hand hygiene in our region. This study also shows that the surgical ICU samples were more contaminated than those of medical ICU (71% vs 37.2%, *p* = 0.004) and the sites frequently touched by both the health-care workers and patients were the most contaminated as the majority of environmental isolates were recovered from floors (42.1%) followed by bed sheets (34.2%) and medical ventilators (10.5%). These findings are in agreement with that of other researchers who reported that this pathogen was isolated from near-patient surfaces, medical equipment, airborne samples and healthcare workers’ hands [5–7, 19].

In the current study, all isolates were resistant to imipinem. Carbapenem-resistance among *A. baumannii* isolates has shown a steady increase in our region since 2001, when it was reported around 23.6% [28] and then increased to 76.19% in 2012–2014 [8]. This can be explained by the excessive use of inadequate empirical antimicrobial treatment including carbapenems, poor infection control practices, poor antimicrobial stewardship governance and widespread dissemination of carbapenem-resistant strains in the community.

In the present study, carbapenem-resistance was mainly attributed to the carriage of the *bla*_*OXA-23-like*_ gene that was present in all isolates (100%).This prevalence is similar to that reported in Turkey (100%) [11] and Egypt (100%) [12] but higher than that observed in Brazil (95.4%) [29], Asian pacific countries (95%) [9], France (82%) [30], South Africa (77%) [31] and Italy (71.7%) [10]. During the past decades, outbreak or sporadic *A. baumannii* clones producing OXA-23 have disseminated around the world [32] but such dissemination has been particularly relevant among Mediterranean countries, where the *bla*_*OXA-23-like*_ gene has replaced previously predominant blaOXA genes such as *bla*_*OXA-24-like*_ and *bla*_*OXA-58-like*_ [33]. In our study, only 2 clinical isolates also harbored the *bla*_*OXA-24-like*_ gene (4.25%) but this gene was not detected among the environmental isolates and all isolates were negative for the *bla*_*OXA-58-like*_ gene. The high prevalence of *bla*_*OXA-23-like*_ gene is probably associated with horizontal gene transfer by mobile genetic elements such as plasmids, transposable elements and integron systems. It has been reported that the spread of *bla*_*OXA-23-like*_ genes is associated with the Tn2006, Tn2007, Tn2008, and Tn2009 transposons, which can be further located on the chromosome or on conjugative plasmids [32, 34, 35].

Likewise, 32.5% of all isolates also presented the *bla*_NDM-1_ gene and this is the first time that NDM-producing *A. baumannii* isolates are reported in Morocco. Our results indicate that NDM-1-producing *A. baumannii* isolates are widely circulating in the hospital environment and they were found in all environmental sampling sites. Moreover, the NDM-1-producing *A. baumannii* isolates were more frequently recovered (*p* = 0.004) from environmental isolates (50%) than from clinical isolates (19.1%).The environmental NDM-1 producing *A. baumannii* isolates have also been reported in Algeria [7] and in China [36]. The high rate of environmental *bla*_NDM-1_ contamination is alarming as the hospital environment may become a potential reservoir for *A. baumannii* isolates carrying NDM-1 which could result in transfer the *bla*_NDM-1_ gene to other bacterial species.

Among clinical isolates, the anal margin samples were identified (*p* = 0.006) as the most common sites of isolation of NDM-1-producing *A. baumannii* isolates. These findings also highlight the role played by fecally-colonized patients as reservoirs for carbapenem-resistant nosocomial *A. baumannii* isolates.

In the current study, the genetic similarity between clinical and environmental isolates was observed in (80/83 = 96.4%) of all isolates, classified into 7 pulsotypes (0001, 0002, 0003, 0005, 0007, 0008 and 0009). These results suggest a dynamic exchange of *A. baumannii* isolates between patients and their environmental surroundings. These pathogens can be transmitted from patient-to-patient, patient to a health care worker, patient to environment and vice versa. In our study, three clinical isolates belonging to the pulsotypes (0004 and 0006) which were not detected in environmental isolates, may be exclusively transmitted through direct contact between an infected or colonized patient and another person or they may come from other environmental reservoirs which are either rare or not identified in our study.

Our results also show that the most frequent pulsotype was PFGE type 0008 (30/83 = 36.1%). Among the two major pulsotypes, clinical isolates were predominant within pulsotype 0007 (16/47 = 34%) while environmental isolates were a majority within pulsotype 0008 (16/36 = 44.4%). Overall, however, both major pulsotypes were closely related (Dice similarity >82%) and contained up to 59% of all isolates, they were found in all sampling sites and it is clear that they have become endemic in this particular setting. The PFGE cluster 0008 corresponds to ST195 (Oxford MLST) which has been previously reported from Asian countries, European nations and Egypt [37–39] while the strains from pulsotype 0007 were assigned to ST 1089(Oxford MLST) which is very rare ST and according to “the profile history for *A. baumannii* MLST (Oxford) database”, the ST 1089 has been found for the first time in India in 2015.

## Conclusion

This study shows that the clonal spread of environmental *A. baumannii* isolates is related to that of clinical isolates recovered from colonized or infected patients. Our results have also shown that OXA-23 is the most common carbapenemase among *A. baumannii* isolates in our hospital but the prevalence of isolates producing both OXA-23 and NDM-1 is also alarming.

Effective control measures are urgently needed to prevent the transmission of endemic lineages of MDR *A. baumannii* and they should take into account the decontamination of the patients’ environmental surroundings.

## Additional file

Additional file 1: Samples sources, collection dates, wards, OXA-type carbapenemases and PFGE of *A. baumannii* isolates. (DOCX 19 kb)

## Abbreviation

DNA: Deoxyribonucleic acid
dNTPs: deoxynucleoside triphosphates
ICU: Intensive care unit
IQR: Interquartile range
MALDI-TOF-MS: Matrix-assisted laser desorption/ionization time-of-flight mass spectrometry
MDR: Multidrug-resistant
MLST: Multi locus sequence typing
PCR: Polymerase chain reaction
PFGE: Pulsed-field gel electrophoresis
ST: Sequence type
UPGMA: Unweighted Pair Group Method with Arithmetic Mean
