# Supplementary material for: Clonal diversity and detection of carbapenem resistance encoding genes among multidrug-resistant Acinetobacter baumannii isolates recovered from patients and environment in two intensive care units in a Moroccan hospital
Source: Antimicrob Resist Infect Control. 2017 Sep 26;6:99. doi: 10.1186/s13756-017-0262-4 (PMC5615474; doi:10.1186/s13756-017-0262-4)
Supplement: Additional file 1: — Samples sources, collection dates, wards, OXA-type carbapenemases and PFGE of A. baumannii isolates. (DOCX 19 kb) [file 13756_2017_262_MOESM1_ESM.docx]

**Supplemented DATA: Samples sources, collection dates, wards, OXA-type carbapenemases and PFGE of A. baumannii isolates (Supplemented DATA)**

| **N°of strain** | **Source** | **Date of isolation** | **Wards** | **site of isolation** | **IMP** | **PFGE pulsotype** | **Carbapenemase genes** |
| --- | --- | --- | --- | --- | --- | --- | --- |
| **1C** | **C** | **30/03/2015** | **S-ICU** | **anal margin** | **R** | **0008** | **OXA-51/OXA-23** |
| **2C** | **C** | **20/04/2015** | **S-ICU** | **anal margin** | **R** | **0005** | **OXA-51/OXA-23** |
| **3C** | **C** | **02/07/2015** | **S-ICU** | **Blood culture** | **R** | **0008** | **OXA-51/OXA-23** |
| **4C** | **C** | **16/03/2015** | **S-ICU** | **mouth** | **R** | **0005** | **OXA-51/OXA-23** |
| **5C** | **C** | **20/04/2015** | **S-ICU** | **anal margin** | **R** | **0007** | **OXA-51/OXA-23** |
| **6C** | **C** | **26/03/2015** | **M-ICU** | **anal margin** | **R** | **0007** | **OXA-51/OXA-23** |
| **7C** | **C** | **17/03/2015** | **S-ICU** | **bronchial aspiration** | **R** | **0007** | **OXA-51/OXA-23** |
| **8C** | **C** | **10/03/2015** | **M-ICU** | **anal margin** | **R** | **0001** | **OXA-51/OXA-23** |
| **9C** | **C** | **30/03/2015** | **S-ICU** | **mouth** | **R** | **0008** | **OXA-51/OXA-23** |
| **10C** | **C** | **21/03/2015** | **M-ICU** | **mouth** | **R** | **0007** | **OXA-51/OXA-23** |
| **11C** | **C** | **21/03/2015** | **M-ICU** | **anal margin** | **R** | **0008** | **OXA-51/OXA-23** |
| **12C** | **C** | **25/03/2015** | **M-ICU** | **anal margin** | **R** | **0008** | **OXA-51/OXA-23/NDM-1** |
| **13C** | **C** | **27/04/2015** | **M-ICU** | **anal margin** | **R** | **0008** | **OXA-51/OXA-23** |
| **14C** | **C** | **13/04/2015** | **M-ICU** | **mouth** | **R** | **0005** | **OXA-51/OXA-23** |
| **15C** | **C** | **04/03/2015** | **M-ICU** | **mouth** | **R** | **0007** | **OXA-51/OXA-23** |
| **16C** | **C** | **24/04/2015** | **S-ICU** | **bronchial aspiration** | **R** | **0007** | **OXA-51/OXA-23/OXA-24** |
| **17C** | **C** | **10/06/2015** | **S-ICU** | **Blood culture** | **R** | **0007** | **OXA-51/OXA-23** |
| **18C** | **C** | **18/06/2015** | **S-ICU** | **protected distal sampling** | **R** | **0007** | **OXA-51/OXA-23/OXA-24** |
| **19C** | **C** | **18/06/2015** | **S-ICU** | **Blood culture** | **R** | **0008** | **OXA-51/OXA-23** |
| **20C** | **C** | **16/05/2015** | **S-ICU** | **anal margin** | **R** | **0001** | **OXA-51/OXA-23** |
| **21C** | **C** | **01/06/2015** | **S-ICU** | **groin** | **R** | **0001** | **OXA-51/OXA-23** |
| **22C** | **C** | **01/06/2015** | **S-ICU** | **groin** | **R** | **0007** | **OXA-51/OXA-23** |
| **23C** | **C** | **01/06/2015** | **M-ICU** | **anal margin** | **R** | **0009** | **OXA-51/OXA-23** |
| **24C** | **C** | **07/05/2015** | **M-ICU** | **mouth** | **R** | **0007** | **OXA-51/OXA-23** |
| **25C** | **C** | **08/04/2015** | **S-ICU** | **anal margin** | **R** | **0001** | **OXA-51/OXA-23/NDM-1** |
| **26C** | **C** | **05/03/2015** | **S-ICU** | **mouth** | **R** | **0001** | **OXA-51/OXA-23/NDM-1** |
| **27C** | **C** | **09/05/2015** | **S-ICU** | **anal margin** | **R** | **0007** | **OXA-51/OXA-23/NDM-1** |
| **28C** | **C** | **18/04/2015** | **M-ICU** | **anal margin** | **R** | **0007** | **OXA-51/OXA-23/NDM-1** |
| **29C** | **C** | **27/04/2015** | **S-ICU** | **anal margin** | **R** | **0007** | **OXA-51/OXA-23/NDM-1** |
| **30C** | **C** | **23/07/2015** | **M-ICU** | **anal margin** | **R** | **0007** | **OXA-51/OXA-23/NDM-1** |
| **31C** | **C** | **23/07/2015** | **S-ICU** | **mouth** | **R** | **0008** | **OXA-51/OXA-23** |
| **32C** | **C** | **23/07/2015** | **M-ICU** | **anal margin** | **R** | **0008** | **OXA-51/OXA-23** |
| **33C** | **C** | **23/07/2015** | **M-ICU** | **protected distal sampling** | **R** | **0006** | **OXA-51/OXA-23** |
| **34C** | **C** | **18/05/2015** | **S-ICU** | **mouth** | **R** | **0008** | **OXA-51/OXA-23** |
| **35C** | **C** | **23/07/2015** | **S-ICU** | **anal margin** | **R** | **0001** | **OXA-51/OXA-23/NDM-1** |
| **36C** | **C** | **23/07/2015** | **M-ICU** | **anal margin** | **R** | **0003** | **OXA-51/OXA-23** |
| **37C** | **C** | **19/03/2015** | **M-ICU** | **groin** | **R** | **0008** | **OXA-51/OXA-23** |
| **38C** | **C** | **06/04/2015** | **S-ICU** | **groin** | **R** | **0008** | **OXA-51/OXA-23** |
| **39C** | **C** | **13/04/2015** | **S-ICU** | **anal margin** | **R** | **0002** | **OXA-51/OXA-23/NDM-1** |
| **40C** | **C** | **05/05/2015** | **M-ICU** | **anal margin** | **R** | **0007** | **OXA-51/OXA-23** |
| **41C** | **C** | **15/05/2015** | **S-ICU** | **anal margin** | **R** | **0009** | **OXA-51/OXA-23** |
| **42C** | **C** | **22/04/2015** | **S-ICU** | **groin** | **R** | **0004** | **OXA-51/OXA-23** |
| **43C** | **C** | **18/05/2015** | **S-ICU** | **groin** | **R** | **0007** | **OXA-51/OXA-23** |
| **44C** | **C** | **20/03/2015** | **M-ICU** | **mouth** | **R** | **0004** | **OXA-51/OXA-23** |
| **45C** | **C** | **02/07/2015** | **S-ICU** | **protected distal sampling** | **R** | **0008** | **OXA-51/OXA-23** |
| **46C** | **C** | **15/05/2015** | **M-ICU** | **anal margin** | **R** | **0001** | **OXA-51/OXA-23** |
| **47C** | **C** | **05/05/2015** | **S-ICU** | **groin** | **R** | **0008** | **OXA-51/OXA-23** |
| **1E** | **E** | **01/04/2015** | **S-ICU** | **bed sheet** | **R** | **0008** | **OXA-51/OXA-23/NDM-1** |
| **2E** | **E** | **01/04/2015** | **S-ICU** | **pillow** | **R** | **0008** | **OXA-51/OXA-23/NDM-1** |
| **3E** | **E** | **01/04/2015** | **S-ICU** | **patient trolley** | **R** | **0008** | **OXA-51/OXA-23/NDM-1** |
| **4E** | **E** | **01/04/2015** | **S-ICU** | **floor** | **R** | **0005** | **OXA-51/OXA-23** |
| **5E** | **E** | **01/04/2015** | **S-ICU** | **intravenous solution stand** | **R** | **0008** | **OXA-51/OXA-23/NDM-1** |
| **6E** | **E** | **01/04/2015** | **S-ICU** | **medical ventilator** | **R** | **0008** | **OXA-51/OXA-23/NDM-1** |
| **7E** | **E** | **04/05/2015** | **M-ICU** | **bed sheet** | **R** | **0008** | **OXA-51/OXA-23/NDM-1** |
| **8E** | **E** | **04/05/2015** | **S-ICU** | **floor** | **R** | **0008** | **OXA-51/OXA-23/NDM-1** |
| **9E** | **E** | **06/05/2015** | **M-ICU** | **floor** | **R** | **0001** | **OXA-51/OXA-23** |
| **10E** | **E** | **06/05/2015** | **M-ICU** | **bed sheet** | **R** | **0008** | **OXA-51/OXA-23/NDM-1** |
| **11E** | **E** | **06/05/2015** | **M-ICU** | **monitor** | **R** | **0002** | **OXA-51/OXA-23/NDM-1** |
| **12E** | **E** | **06/05/2015** | **S-ICU** | **floor** | **R** | **0005** | **OXA-51/OXA-23** |
| **13E** | **E** | **06/05/2015** | **S-ICU** | **bed sheet** | **R** | **0008** | **OXA-51/OXA-23/NDM-1** |
| **14E** | **E** | **06/05/2015** | **M-ICU** | **floor** | **R** | **0005** | **OXA-51/OXA-23** |
| **15E** | **E** | **06/05/2015** | **M-ICU** | **patient trolley** | **R** | **0007** | **OXA-51/OXA-23** |
| **16E** | **E** | **06/05/2015** | **S-ICU** | **bed sheet** | **R** | **0007** | **OXA-51/OXA-23** |
| **17E** | **E** | **06/05/2015** | **S-ICU** | **bed sheet** | **R** | **0002** | **OXA-51/OXA-23/NDM-1** |
| **18E** | **E** | **11/05/2015** | **S-ICU** | **bed sheet** | **R** | **0008** | **OXA-51/OXA-23/NDM-1** |
| **19E** | **E** | **11/05/2015** | **S-ICU** | **floor** | **R** | **0008** | **OXA-51/OXA-23/NDM-1** |
| **20E** | **E** | **11/05/2015** | **S-ICU** | **medical ventilator** | **R** | **0005** | **OXA-51/OXA-23** |
| **21E** | **E** | **11/05/2015** | **M-ICU** | **medical ventilator** | **R** | **0008** | **OXA-51/OXA-23/NDM-1** |
| **22E** | **E** | **11/05/2015** | **M-ICU** | **floor** | **R** | **0008** | **OXA-51/OXA-23/NDM-1** |
| **23E** | **E** | **11/05/2015** | **M-ICU** | **bed sheet** | **R** | **0009** | **OXA-51/OXA-23** |
| **24E** | **E** | **11/05/2015** | **S-ICU** | **bed sheet** | **R** | **0008** | **OXA-51/OXA-23** |
| **25E** | **E** | **11/05/2015** | **S-ICU** | **floor** | **R** | **0008** | **OXA-51/OXA-23/NDM-1** |
| **26E** | **E** | **13/05/2015** | **S-ICU** | **bed sheet** | **R** | **0008** | **OXA-51/OXA-23/NDM-1** |
| **27E** | **E** | **13/05/2015** | **M-ICU** | **medical ventilator** | **R** | **0007** | **OXA-51/OXA-23/NDM-1** |
| **28E** | **E** | **18/05/2015** | **S-ICU** | **bed sheet** | **R** | **0003** | **OXA-51/OXA-23** |
| **29E** | **E** | **18/05/2015** | **S-ICU** | **floor** | **R** | **0003** | **OXA-51/OXA-23** |
| **30E** | **E** | **18/05/2015** | **M-ICU** | **bed sheet** | **R** | **0003** | **OXA-51/OXA-23** |
| **31E** | **E** | **18/05/2015** | **M-ICU** | **floor** | **R** | **0009** | **OXA-51/OXA-23** |
| **32E** | **E** | **18/05/2015** | **S-ICU** | **bed sheet** | **R** | **0009** | **OXA-51/OXA-23** |
| **33E** | **E** | **18/05/2015** | **S-ICU** | **floor** | **R** | **0009** | **OXA-51/OXA-23** |
| **34E** | **E** | **18/05/2015** | **S-ICU** | **floor** | **R** | **0003** | **OXA-51/OXA-23** |
| **35E** | **E** | **06/05/2015** | **M-ICU** | **floor** | **R** | **0009** | **OXA-51/OXA-23** |
| **36E** | **E** | **13/05/2015** | **S-ICU** | **bed sheet** | **R** | **0009** | **OXA-51/OXA-23** |
